# Prevalence, Response to Cysticidal Therapy, and Risk Factors for Persistent Seizure in Indian Children with Neurocysticercosis

**DOI:** 10.1155/2017/8983958

**Published:** 2017-01-10

**Authors:** Animesh Kumar, Anirban Mandal, Sheela Sinha, Amitabh Singh, Rashmi Ranjan Das

**Affiliations:** ^1^Department of Pediatrics, All India Institute of Medical Sciences, New Delhi 110029, India; ^2^Department of Pediatrics, Sitaram Bhartia Institute of Science and Research, New Delhi 110016, India; ^3^Department of Pediatrics, Patna Medical College and Hospital, Patna 800004, India; ^4^Department of Pediatrics, Chacha Nehru Bal Chikitsalaya, New Delhi 110031, India; ^5^Department of Pediatrics, All India Institute of Medical Sciences, Bhubaneswar 751019, India

## Abstract

*Background.* Neurocysticercosis (NCC) is the commonest cause of childhood acquired epilepsy in developing countries. The use of cysticidal therapy in NCC, except “single lesion NCC,” is still debated in view of its doubtful usefulness and potential adverse effects.* Methods.* Children presenting with first episode of seizure or acute focal neurological deficit without fever were screened for NCC and received appropriate therapy (followup done for 1 year to look for the response and side effects).* Results.* The prevalence of NCC was 4.5%. Most common presenting feature was generalized seizure and commonest imaging finding was single small enhancing lesion in the parietal lobe. Abnormal EEG and CSF abnormalities were found in almost half of the children. The response to therapy was very good with infrequent recurrence of seizure and adverse effects of therapy were encountered rarely. No risk factors for persistent seizure could be identified.* Conclusion.* Present study shows that the response to cysticidal therapy is very good in NCC as seizure recurrence was observed in only 5%, 4.2%, and 4.2% of cases at 3-month, 6-month, and 1-year followup. Adverse effects of therapy were observed in 20% of cases during therapy but they were mild and self-limiting.

## 1. Background

Neurocysticercosis (NCC), the commonest parasitic infection of the nervous system, is also the most common cause of acquired epilepsy in childhood [1]. In a systematic review, 29% of epileptics were found to have NCC [2]. NCC is endemic in most developing countries of Asia, Latin America, and Central and South Africa [3]. A seroprevalence study from Chandigarh, India, reported anticysticercus antibodies in 17.3% [4] while seroprevalence among the healthy blood donors from Pondicherry, India, was 6.5% using both antigen and antibody detection methods [5]. In a WHO study conducted in rural pig-farming community of UP, India, the prevalence of Taeniasis was 18.6% and epilepsy was found in 5.8% and 48.3% of them had NCC [6].

There is no single therapeutic approach to NCC. The approach depends upon the number and location of lesions, cysts' viability, and host's immune response to the parasite [7]. A combination of symptomatic and cysticidal therapy is usually used. Of the later, albendazole is superior to praziquantel. The use of cysticidal therapy has been questioned, as it destroys the cysts but do not modify the disease course [8]. Based on the available literature, the American Academy of Neurology advised the use of cysticidal therapy with albendazole in cases of NCC, as it was found to be associated with both reduction of long-term seizure and resolution of the lesions [9].

With this background, we conducted a prospective, observational study to find prevalence of NCC, its response to cysticidal therapy, and adverse effects of therapy and also to find the risk factors for persistent seizure in Indian children with NCC.

## 2. Methods

Children between 1 year to 14 years of age visiting the pediatric outpatient department (OPD) and pediatric emergency of a tertiary care hospital in Northern India over 2 years period (May 2012 to August 2014) with first episode of seizure or acute focal neurological deficit were screened. Ethical clearance was obtained from the Institute Ethics Committee. A written informed consent was taken from either of the parents/legal guardians. Patients were excluded if they had fever, known neurological illness, or epilepsy with known etiology (e.g., structural brain anomaly), were critically sick (respiratory failure, cardiovascular instability), and already received antihelminthic (albendazole or praziquantel) or steroid therapy. Patients who were found to have an alternate diagnosis or only calcified NCC or ocular cysticercosis after enrolment were also excluded. Therefore, cases of NCC in vesicular stage, colloid stage, and granular nodular stage, either parenchymal or extraparenchymal, were finally included. Patients' demographic data, presenting illness, past history, family history of seizure/similar illness, dietary history, treatment history, history of exposure to animals, household contact with NCC, and socioeconomic status were recorded. General physical and detailed nervous system examination including direct ophthalmoscopy was done for all the patients.

A complete blood count, liver and renal function tests, mantoux test, chest X-ray, contrast enhanced computed tomography (CT) scan of brain were obtained in all the patients. Magnetic resonance imaging (MRI) of brain (in cases with normal or doubtful CT scan findings), cerebrospinal fluid (CSF) examination, stool microscopic examination for evidence of Taenia solium, Taenia solium antibody in serum and CSF (ELISA kit; UB-Magiwell Cysticercosis Kit™), and EEG were done. Diagnosis was based on clinical picture and radiological findings highly suggestive of neurocysticercosis.

All the children were treated with albendazole at a dose of 15 mg/kg/day (maximum 800 mg/day) in 2 divided doses for 28 days along with dexamethasone at a dose of 0.15 mg/kg/dose (maximum 4 mg/dose) every six hours (started 2 days before albendazole and continued for 3 days along with albendazole) for a total duration of 5 days, irrespective of the number of NCC. Children also received antiepileptic for seizure control. Children were followed up at 1-week, 2-week, 1-month, 3-month, 6-month, and 1-year period after starting the cysticidal therapy. At each followup, frequency of seizure occurrence, symptomatic improvement/worsening of symptoms (other associated features like raised intracranial tension, focal neurological deficits, encephalopathy, and visual problems), and adverse drug reactions (vomiting, abdominal pain, rash, and jaundice) were noted. Laboratory tests were repeated at 1 week, 2 weeks, and the end of therapy (i.e., at 1 month). Various presenting features and laboratory parameters between children who had a persistence/recurrence of seizure at 1 year of followup and those who did not have seizure at 1 year were compared in an attempt to find out the possible risk factors. All the data were entered in a predesigned performa and managed in Microsoft excel spreadsheet. The data was expressed as number and percentage (for categorical variables) or mean with standard deviation (for continuous variable). Statistical analysis was done using Stata 11 software (Statacorp, TX, USA). Student's *t* test and Chi square test were used for determining the *P* value (<0.05 was taken as significant).

## 3. Results

**Figure 1:**
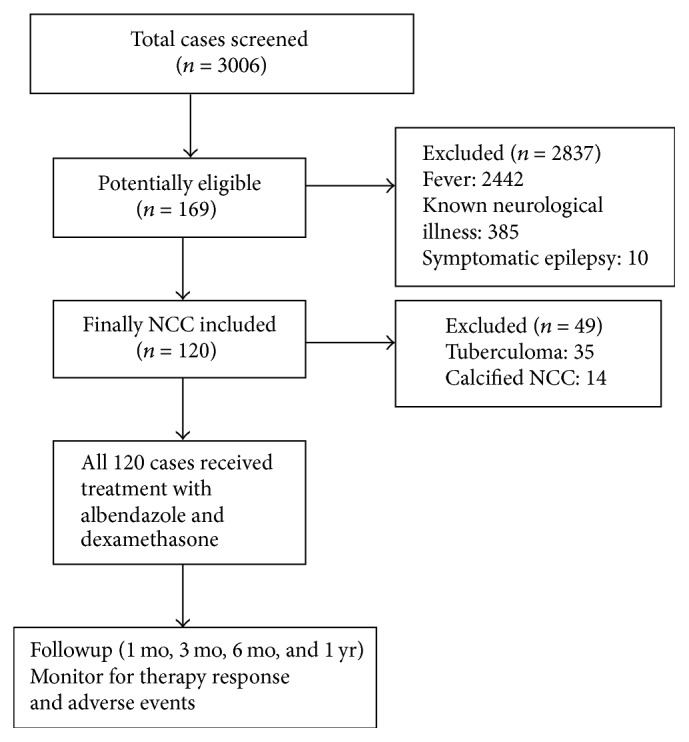
Flow diagram showing recruitment of cases into the study.

A total of 3006 patients were screened. Of 169 initially included, 35 turned out to be tuberculoma and 14 had calcified cysts, so they were further excluded. Finally 120 cases of NCC in vesicular stage, colloid stage, and granular nodular stage, either parenchymal or extra-parenchymal according to neuroimaging, were included for treatment and further followup (Figure 1).

The baseline characteristics of these children have been described (Table 1). Most of the patients were in the 10–14 years of age group, and around 56% were male. Commonest presenting complaint was seizure with generalized seizure in 92 cases (86.7%). Commonest type of lesion observed on neuroimaging was a solitary lesion in brain (67.5%), while none of the children had >20 lesions. In majority (85%) of the cases, lesions were located in the brain parenchyma only, and rests were having lesions both in brain parenchyma and ventricles. None of the cases were noted to have isolated ventricular or cisternal lesions (Table 2). Amongst the parenchymal lesions, parietal lobe was the most common site of involvement (79%), either isolated or along with other site of involvement followed by frontal lobe in around 15% cases. No children were noted to have associated extracranial cysticercosis. Thirty-nine children had “multiple lesion” NCC; 30 (76.9%) of them had raised ICT, and 4 (10.3%) had focal neurological deficits. All the 18 cases with intraventricular NCC had more than 5 NCC lesions with 10 cases having 5–10 lesions and 8 cases with >10 lesions.

Serum Taenia solium antibodies were tested in 43 children and a positivity rate of 65.1% was observed (Table 3). CSF antibodies against T solium was done in 20 children only but it revealed a very high positivity (90%) (Table 4).

Therapy, treatment outcome, appearance of new symptoms, and side effects of therapy in these children on followup has been described (Table 5). Recurrence of seizure was observed in 28 children over 1-year period, and only 5 children had persistent seizure at 6 months and also at 1-year followup. Repeat CT scan revealed calcified lesions (data not shown). The antiepileptic drugs were continued in these cases. Worsening of preexisting symptoms was mostly in the form of worsening preexisting raised intracranial pressure and headache. One child developed pseudoseizure after 6 months of therapy. The number of children who had developed new symptoms attributable to NCC (e.g., headache, features of raised intracranial tension, focal neurological deficits, encephalopathy, and visual problems) during 1 year of followup was very small. Regarding the adverse events, 2 cases developed new onset raised intracranial tension after 1 week and 3 cases after 2 weeks, respectively; cysticidal therapy was stopped in these 5 cases. Eleven cases developed gastrointestinal symptoms (abdominal pain, nausea, and vomiting) during the course of albendazole therapy. They were managed conservatively and did not require stopping of therapy. Three children developed elevation of transaminases (>2 times of upper limit of normal) after 1 week of therapy requiring temporary stoppage of therapy; they normalized over next 2 weeks. Neutropenia occurred in 2 cases at 2 weeks and in 6 cases at 4 weeks requiring temporary stoppage of albendazole therapy. They were also followed up with serial blood counts and improved over next 2 weeks.

While trying to find out any risk factors that could account for persistence of seizure at 1 year, various parameters were compared between children having persistence of seizure at 1 year and those in whom seizure subsided (Table 6). But none of the compared variables were found to be statistically significant between the two groups.

## 4. Discussion

In this hospital based, prospective study, the prevalence of NCC was 4.5% in children presenting with acute onset seizure/focal neurological deficit. The 10–14-year age group was most commonly affected. Similar findings are reported in previous studies as well [6, 10]. This could be explained by the fact that children share the family diet usually late, and though symptoms may occur as early as months after primary infestation, it usually occurs 2–6 years after exposure [11]. No predilection of sex could be identified. NCC was found to be more prevalent in the children belonging to lower socioeconomic strata. This could be explained by poorer standards of living and lack of hygiene that goes hand in hand with poverty. Another postulate is that, with pork being a cheaper meat, it is more consumed by poor, and eating pork has been found to be a risk factor for the disease [6].

Seizure was the commonest (86.7%) presenting feature with generalized seizure (76.7%) being more common than focal. This may seem to be in contrast to the common belief and also most of the previously reported studies from India and abroad [12–14]. But two large case series published recently from Nepal [15] and India [16] cited incidences of generalized seizure at presentations 52% and 65%, respectively. But this should be interpreted cautiously, as the chances of focal seizure leading to secondary generalization being interpreted as GTCS cannot be ruled out as the seizure seminology was discerned from parental history and the onset may have been missed by them. Features suggestive of raised intracranial pressure and focal neurological deficit are less common in children compared to adults. Our figures of 28.3% and 4% are similar to the 30% and 8% reported by another study [12].

Around 11% children had peripheral blood eosinophilia (absolute eosinophil count >500/mm^3^); therefore, it was of little benefit in the diagnosis. Only 5.8% children with NCC were found to be excreting ova of Taenia solium in their stool. The stool positivity rates for T solium were always found to be very scarce in previous pediatric studies [12, 15], but interestingly, a recent study from India [17] has reported a very high rate (44.7%) of isolation of T solium ova in stool. CSF examination was normal in most (45%) cases. The commonest abnormal finding was noted to be pleocytosis followed by raised protein. These findings were also partially congruent with previously conducted pediatric and adult studies [11, 18]. Few (13.3%) cases showed CSF eosinophilia which is considered a suggestive finding towards the diagnosis of CNS helminthic infection though not specific of neurocysticercosis [19]. An abnormal EEG was observed in 56% of cases, the commonest abnormality being sharp wave and spikes followed by nonspecific background abnormalities. However, EEG has minor relation to the symptoms and CT lesions in NCC [20].

Commonest radiological pattern of NCC was a solitary lesion (67.5%). Parietal lobe was the most common site of involvement (79%) followed by frontal lobe (15%). Commonest site of involvement was brain parenchyma, either alone or in combination with ventricle. These are consistent with findings from other studies [12, 15–18].

Only 65% of 40 cases were found positive for serum antibody in our study, whereas CSF antibody positivity rates of 90% were observed in 20 children tested for the same. Serum ELISA has always been associated with poor sensitivity [1]. Other possible explanations are that false negative serum antibody results by ELISA may be higher in patients with only parenchymatous disease, especially with single lesions [1]. False negative results in serum with positive CSF serology may also occur because of local production of antibodies without a parallel increase in titres in the peripheral blood [21]. On the other hand, false positive results in immune tests are sometimes seen in individuals from endemic areas because of contact with the parasite without development of the disease [1].

Seizure recurrence was observed in 6 (5%), 5 (4.2%), and 5 (4.2%) cases at 3-month, 6-month, and 1-year followup, respectively. These rates have been variable being high (10% and 13% at 3 months and 6 months, resp.) [22] and low (4.8% at 1 year) [23]. On the other hand, a more recent study from India reported a very high rate of seizure recurrence (23.7%) at 2-year followup [17]. None of our children had persistence of any other symptoms attributable to NCC at the end of 1-year followup. Only one child developed pseudoseizure, which would be unrelated to NCC.

A follow-up CT scan was performed only in those with recurrence of seizure (28), worsening of preexisting symptoms (13), or new neurological symptoms (2). Among the children with recurrence of seizure (12) or worsening of preexisting symptoms (11) or development of new symptoms (1) during therapy 5 children were found to have raised ICP. Notably all of them had 2–5 parenchymal NCC (no intraventricular NCC) and only had evidence of perilesional edema with no evidence of hydrocephalus. All of them responded well to short course (7 days) of dexamethasone at a dose of 0.15 mg/kg/dose q 6 hourly along with osmotherapy (mannitol and/or hypertonic saline) and other supportive measures with temporary cessation of cysticidal therapy.

All the patients in whom a CT scan was performed at 3 months and beyond showed complete calcification of NCC. Calcification in NCC is a common long-term outcome with observed rates of calcification in pediatric NCC varying widely from 6.2% to as high as 60% [24]. Interestingly, cysticidal therapy was found to have no significant effect in the calcification of solitary parenchymal NCC [25, 26]. A retrospective analysis of solitary parenchymal NCC from India [24] reported that the risk of breakthrough seizures was significantly higher in calcified lesions on follow-up scan when compared to normal scans or persisting lesions. The same has been echoed in another prospective study involving both children and adults [27].

There are very few pediatric studies which document adverse effects of albendazole therapy other than neurological symptoms [24]. Gastrointestinal, hepatic, and hematological side effects of albendazole are well known, and guidelines for monitoring of patients on long-term therapy advise laboratory monitoring twice weekly [28]. In our study, gastrointestinal side effects were observed in 11 (9.2%) children, elevated transaminases in 3 (2.5%), and neutropenia in 8 (6.7%) requiring temporary cessation of therapy. Both hepatic and hematological abnormalities normalized on followup within few weeks. Variable gastrointestinal side effects have been observed in various studies (from 6% to 26%) [29, 30].

Sharma et al. [27] found that apart from calcification of the lesions family history of seizures, serial seizures, and presence of EEG abnormalities is to be associated with increased risk of recurrence of seizures. While trying to find out risk factors for persistence of seizure at 1 year in our population, none of the perceived risk factors (e.g., mean age at onset of seizure, partial seizure, >5 lesions in brain, and EEG abnormality) were found to be important. Similar findings were observed by others [17, 23], who also failed to find any risk factors for persistence of seizures in children with NCC.

The present study has many strengths: longitudinal design, robust methodology, and prolonged clinical and laboratory followup of the cases after therapy to look for response as well as adverse effects which is rarely described in previous pediatric studies. Following are the limitations. First, neuroimaging and EEG were not repeated in the children after treatment in the followup. It would have revealed response to antihelminthic therapy and also helped in deciding on the duration of antiepileptic. Then again, repeating a CT scan definitely increases radiation exposure to the developing brain of these children. MRI though does not involve radiation; it is associated with need for prolonged sedation or general anesthesia and the associated risk of the same. The cost of the above mentioned procedures is also to be kept in mind in the context of a resource poor setting like India. Second, being a tertiary care hospital based study, the participants may not be representative of the population intended.

## 5. Conclusion

Present study shows that the response to cysticidal therapy is very good in NCC with infrequent adverse effects.

## Figures and Tables

**Table 1:** Baseline characteristics and investigations in children with NCC (*N* = 120).

Characteristics	Children with NCC (*N* = 120)
*Age distribution*	
1–4 years	8 (6.7%)
5–10 years	48 (40%)
11–14 years	64 (53.3%)

*Boys*	67 (55.8%)

*Socioeconomic status (modified Kuppuswamy scale)*	
Upper	8 (6.7%)
Upper-middle	25 (20.8%)
Lower-middle	25 (20.8%)
Upper-lower	40 (33.3%)
Lower	22 (18.3%)

*Nonvegetarian*	77 (64.2%)

*Symptoms/signs*	
Seizure	104 (86.7%)
Raised intracranial pressure	34 (28.3%)
Altered sensorium	32 (26.7%)
Focal neurological deficit	8 (6.7%)

*Peripheral blood eosinophilia*	13 (10.8%)

*Stool examination positive for Taenia solium ova*	7 (5.8%)

*CSF findings*	
Pleocytosis	56 (46.7%)
Elevated protein	41 (34.2%)
Low glucose (<1/2 of concurrently measured blood glucose)	32 (26.7%)
Eosinophils in centrifuged sediment	16 (13.3%)
Normal CSF	54 (45%)

*Abnormal EEG*	56 (46.7%)

**Table 2:** Neuroimaging findings in children with NCC (*N* = 120).

Neuroimaging findings	Number (%)
*Number of lesions*	
Single	81 (67.5%)
2–5	17 (14.2%)
6–10	14 (11.7%)
11–20	8 (6.7%)
>20	0

*Location in brain*	
Parenchyma only	102 (85%)
Combined (parenchyma and ventricular)	18 (15%)
Cistern only	0
Ventricles only	0

*Location of the parenchymal lesions*	
Parietal lobe	79%
Frontal lobe	15%
Temporal lobe	0
Occipital lobe	6%

**Table 3:** Serum T solium antibodies by ELISA in children with neurocysticercosis (*N* = 43).

Results	Number	Percentage
Positive	28	65.1%
Negative	15	34.9%

**Table 4:** CSF T. solium antibodies in children with neurocysticercosis (*N* = 20).

Results	Number	Percentage
Positive	18	90.0%
Negative	2	10.0%

**Table 5:** Therapy, treatment outcome, appearance of new symptoms, and side effects of therapy in children with neurocysticercosis.

Follow-up duration	*N*	Seizure	Worsening of other preexisting symptoms	New symptoms	Side effects of medication
1 week	114	0	2	0	5
2 weeks	109	4	5	0	9
1 month	105	8	4	1	10
3 months	98	6	2	1	NA
6 months	90	5	0	0	NA
1 year	81	5	0	0	NA

**Table 6:** Comparison between patients with and without persistence of seizure at 1 year.

Parameters	Patients with persistence of seizure at 1 year (*n* = 5)^*∗*^	Patients without persistence of seizure at 1 year (*n* = 76)^*∗*^	*P* value
Mean age of onset (years)	9.6 ± 2.9	10.8 ± 3.2	0.41
Male gender	4 (75%)	42 (55.3%)	0.53
Partial seizure at presentation	3 (60%)	22 (29%)	0.33
Lesions >5 in neuroimaging	2 (40%)	17 (22.4%)	0.72
EEG abnormality at presentation	3 (60%)	40 (52.6%)	0.75

^*∗*^A total of 81 patients could be followed up for 1 year.
